# 318. Beta-lactam Therapeutic Drug Monitoring Improves Attainment of Target Drug Levels in Critically Ill Patients

**DOI:** 10.1093/ofid/ofad500.389

**Published:** 2023-11-27

**Authors:** Sara Ausman, Shienna Braga, Natalie T Hagy, Christina G Rivera (O'Connor), Lindsay Moreland-Head, Wessel Rebecca, Omar M Abu Saleh, Andrew D Rule, ognjen Gajic, Erin F Barreto

**Affiliations:** Mayo Clinic Health System - Eau Claire, Eau Claire, Wisconsin; Mayo Clinic Hospital - Rochester, Rochester, Minnesota; Mayo Clinic, Rochester, Minnesota; Mayo Clinic, Rochester, Minnesota; Indiana University School of Medicine - West Lafayette, West Lafayette, Indiana; Mayo Clinic, Rochester, Minnesota; Mayo Clinic Rochester, Rochester, Minnesota; Mayo Clinic, Rochester, Minnesota; Mayo, Rochester, Minnesota; Mayo Clinic, Rochester, Minnesota

## Abstract

**Background:**

Critically ill patients, particularly those treated with extracorporeal devices or at extremes of weight, experience pharmacokinetic variability which can compromise beta-lactam (BL) antibiotic target attainment and treatment response. Therapeutic drug monitoring (TDM) for BL antibiotics can improve precision pharmacotherapy but has had limited implementation. The objective of this study was to evaluate the frequency of BL target attainment among adult intensive care unit (ICU) patients who underwent TDM.

**Methods:**

This observational study evaluated adults treated with cefepime, piperacillin/tazobactam or meropenem in ICUs at the Mayo Clinic in Rochester, Minnesota who underwent TDM from June-September 2022. During the study timeframe, multidisciplinary teams were encouraged to perform BL TDM on patients requiring extracorporeal membrane oxygenation, continuous kidney replacement therapy, or at an extreme of body weight (weight < 40 kg or >120 kg or BMI < 18 kg/m^2^ or > 40 kg/m^2^). Percentage of patients with initial serum trough concentrations within the therapeutic range was calculated (Table 1).

**Figure:**
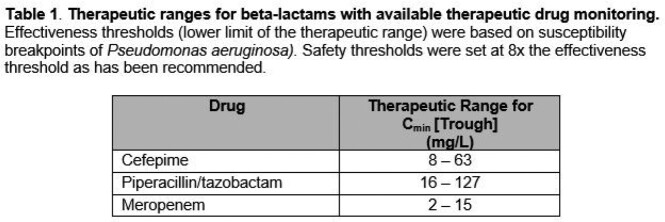

**Results:**

During the study timeframe, 59 trough concentrations were performed on critically ill patients, of which 5 were excluded due to uninterpretable results. Of the remaining 54 BL trough concentrations analyzed, 49 (91%) were initial troughs during the antibiotic course, and 5 (9%) were repeat troughs. Thirty-six (73%) of the initial trough concentrations were within the therapeutic range. Among the trough concentrations outside the therapeutic range, 10 (77%) were too high, and 3 (23%) were too low (Table 2). Dose adjustment occurred in 19 (39%) of initial trough evaluations, including 6 which were adjusted despite a level within the therapeutic range. All of the repeat trough concentrations drawn during the same antibiotic course were within the therapeutic range (n = 5/5; 100%).

**Figure:**
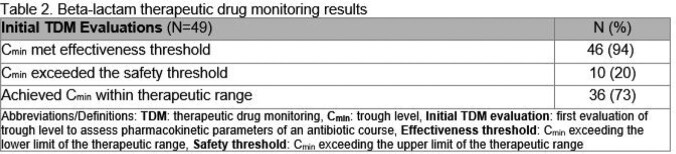

**Conclusion:**

One-fourth of initial BL trough concentrations were outside of the therapeutic range. BL levels outside the therapeutic range prompted dose adjustment to improve the potential for effectiveness or limit the potential for toxicity. BL TDM appears to be a promising strategy to enhance precision pharmacotherapy in the critically ill.

**Disclosures:**

**Sara Ausman, PharmD**, Gilead: Honoraria **Christina G. Rivera (O'Connor), Pharm.D**, Gilead Sciences: Advisor/Consultant|Gilead Sciences: Board Member|Gilead Sciences: Grant/Research Support|Gilead Sciences: Honoraria

